# Psychological Health, Sleep Quality, Behavior, and Internet Use Among People During the COVID-19 Pandemic: A Cross-Sectional Study

**DOI:** 10.3389/fpsyt.2021.632496

**Published:** 2021-03-31

**Authors:** Muhammed Elhadi, Ahmed Alsoufi, Ahmed Msherghi, Entisar Alshareea, Aimen Ashini, Taha Nagib, Nada Abuzid, Sanabel Abodabos, Hind Alrifai, Eman Gresea, Wisal Yahya, Duha Ashour, Salma Abomengal, Noura Qarqab, Amel Albibas, Mohamed Anaiba, Hanadi Idheiraj, Hudi Abraheem, Mohammed Fayyad, Yosra Alkilani, Suhir Alsuwiyah, Abdelwahap Elghezewi, Ahmed Zaid

**Affiliations:** ^1^Faculty of Medicine, University of Tripoli, Tripoli, Libya; ^2^Faculty of Medicine, Al-Zawia University, Zawia, Libya; ^3^Faculty of Medicine, Libyan International Medical University, Benghazi, Libya; ^4^Faculty of Medicine, University of Benghazi, Benghazi, Libya; ^5^Faculty of Medicine, Sabha University, Sabha, Libya; ^6^Internal Medicine, Misurata Central Hospital, Misurata, Libya

**Keywords:** COVID-19, depression, anxiety, SARS-CoV-2, sleep, civil war, pandemic

## Abstract

**Background:** The COVID-19 pandemic has led to an increase in the risk of suicide, uncertainty, mental stress, terror, annoyance, weariness, financial issues, and frustration. We aim to determine the prevalence of insomnia, depressive and anxiety symptoms, and their associated factors among Libyan populations during the COVID-19 pandemic and the civil war.

**Methods:** An online cross-sectional survey was conducted among the Libyan population between July 18 and August 23, 2020. The data collected included basic demographic characteristics, level of education, employment status, COVID-19-related questions, and questions about abuse and domestic violence. This study assessed the psychological status of participants who were screened for anxiety symptoms using the seven-item Generalized Anxiety Disorder scale (GAD-7). Depressive symptoms were also screened for using the two-item Patient Health Questionnaire (PHQ-2) and the Insomnia Severity Index (ISI). Binomial logistic regression was used to predict the probability of insomnia, anxiety and depressive symptoms.

**Results:** A total of 10,296 responses were recorded. Among the participants, 4,756 (46.2%) obtained a cut-off score of ≥ 3 which indicated depressive symptoms. For anxiety, 1,952 participants (19%) obtained a cut-off score of ≥ 15, which indicated anxiety symptoms. For the ISI, the mean (SD) was 11.4 (6.1) for the following categories: no clinical insomnia (0–7) 3,132 (30.4%), sub-threshold insomnia (1–7) 3,747 (36.4%), moderate severity clinical insomnia (8–14) 2,929 (28.4%), and severe clinical insomnia (15–21) 488 (4.7%). Logistic regression analysis showed that depressive symptoms were statistically associated with age, marital status, education level, occupational category, financial problems during the COVID-19 pandemic, health status, having a COVID-19 infection, current health status, suicide ideation, abuse or domestic violence, and lockdown compliance (*p* < 0.05). The regression analysis revealed a statistically significant association between anxiety symptoms and age, education level, occupational status, financial problems during the COVID-19 pandemic, having a COVID-19 infection, health status, suicide ideation, abuse or domestic violence, and lockdown compliance (*p* < 0.05). The regression analysis revealed a statistically significant association between insomnia and all study variables with the exception of age, educational level, and occupational status (*p* < 0.05).

**Conclusion:** Confronted with the COVID-19 outbreak, the Libyan population exhibited high levels of psychological stress manifested in the form of depressive and anxiety symptoms, while one-third of the Libyan population suffered from clinical insomnia. Policymakers need to promote effective measures to reduce mental health issues and improve people's quality of life during the civil war and the COVID-19 pandemic.

## Introduction

The emergence of coronavirus disease 2019 (COVID-19) around the world resulted in more than 105 million cases and over 2.3 million deaths in more than 120 countries around the world by 6th February 2021 (22). The COVID-19 pandemic became a global crisis, and it has resulted in enormous medical and socio-economic burdens worldwide, which may lead to economic recession due to substantial job losses and the cessation of major facilities around the world (23). Since the outbreak of COVID-19 in Libya on March 24, 2020, the number of cases reached more than 123,000, with more than 1,936 deaths as of 6th February 2021 (24, 25). As the pandemic evolved, between 300 and 500 new daily cases were detected, in a country that continues to suffer from an ongoing civil war and a health crisis that is further aggravated by a healthcare system that is ill prepared to fight the COVID-19 pandemic (26, 27).

Because of its accelerated spread, high incidence rate, and mortality rate, the COVID-19 pandemic is the largest public health crisis in decades (28). It is an extremely infectious viral disease caused by severe acute respiratory syndrome coronavirus 2 (SARS-CoV-2), the symptoms of which include fever, cough, nausea, breathing difficulties in humans, and transmission via aerosol droplets, human body touching, and surface contacts (1, 2). To reduce the transmission of COVID-19, preventative techniques included restricting physical interaction to the point of home lockdowns and curfews, reducing the number of passengers on public transport, restrictions, banning public gathering events and places, and social distancing measures (3, 4). In addition, measures such as the wearing of facemasks and regular handwashing aimed to reduce viral transmission among populations (5, 6).

Hopelessness, rage, lack of motivation, fear of death, difficulties falling and staying asleep, loss of appetite, depression, and anxiety were the most prevalent behavioral and psychosocial symptoms observed during the COVID-19 pandemic in this particular region of the world (7, 8). Furthermore, the COVID-19 pandemic led to an increase in the risk of suicide, uncertainty, mental stress, terror, annoyance, weariness, financial issues, and frustration (9). During this stressful time, people experienced behavioral and psychological problems throughout the pandemic, which contributed to poor self-hygiene, sleep disturbances, appetite changes, and a reduced willingness to follow the orders of the health authorities, which in turn increased the vulnerability to infection (10–12).

Several researchers examined the psychological status of the general population during the COVID-19 pandemic. In China (13), 53.8% reported that they experienced a moderate or severe psychological impact of the outbreak. In Italy (14), 38% of the general population reported that they experienced a form of psychological distress, while another study conducted in Spain (15) revealed that, among the general population, 18.7% experienced depression and 21.6% experienced anxiety symptoms. Another study conducted in Australia (16) found that 78% of participants reported a decline in their mental well-being after the outbreak. A national assessment study of at-risk populations in France (17) found that 38.06% experienced psychological distress. Several factors have been identified as increasing this psychological distress, such as financial issues, domestic abuse, job loss or work-related changes, sleep disturbances, and several socioeconomic factors. To our knowledge, however, no studies have been carried out in African countries or in countries that are suffering from a civil war, such as Libya. Therefore, there is a need to conduct large-scale population-based studies to assess the effects of the pandemic effect on the general population.

Previous research has reported the occurrence of sleep problems after significant traumatic incidents, such as a pandemic or a natural disaster. COVID-19 has caused tremendous tension, anxiety, and depression. It has adversely affected the population's well-being, and resulted in social alienation, financial problems, and job disruption (18, 19). Individuals whose lives have been significantly disrupted are more likely to experience sleep problems and disrupted circadian cycles. During such times, regulated sleep patterns are essential so that individuals can be in a better position to deal with the instability brought about by the global crisis (20). However, few studies have been conducted to determine the prevalence and risk factors for insomnia and psychological symptoms during the COVID-19 pandemic among the general population. Previous reports focused on healthcare workers or on the early phase of the pandemic in the case of specific populations. For example, research was conducted in China between February 5, 2020, and February 23, 2020 involving 5,461 individuals (20, 21), and in Italy, where a study of the lockdown was carried out among 1,310 people (29). Therefore, there is a need to assess sleep disturbances and associated psychological symptoms in other countries.

Another concern that arose during the COVID-19 pandemic lockdown was the excessive use and misuse of the Internet, which has been reported in previous literature. The rising prevalence of smartphones and technology devices, and the fact that it is now routine for people to spend excessive amounts of time on the Internet, may have negative consequences, such as an increase in sleep latency and a reduction in sleep duration (30, 31). Therefore, problematic Internet use and excessive use of social media can result in psychosocial and sleep problems, especially among young people who used the Internet excessively during the COVID-19 confinement (32). A previous study conducted among 1,242 Wuhan residents in China found that 30.0% of participants had a sleep disorder. The study also revealed that people who use the Internet several times a day have more sleep disorders than those who use it less often (33). Excessive technology use or its addiction leads to extreme psychological effect. A study by Mamun et al. (34) presented suicidality cases as of gaming related issues (34), similar incidences were reported prior to the pandemic (35, 36). Therefore, internet use and digital entertainment consumption during the lockdown can increase risk of catastrophic events among general population and assessing changes in behavior and internet use during this difficult time is essential to implement behavioral and public health policies in order to mitigate the lockdown negative consequences.

In this study, we aimed to determine the prevalence of depressive and anxiety symptoms among Libyan populations. Moreover, we aimed to provide an overview of the prevalence of insomnia and associated factors, and to carry out a preliminary analysis of Internet use, behavioral changes, and sleep disorders among Libyan populations during the COVID-19 pandemic and the civil war.

## Methods

### Study Design, Setting, and Study Period

An online cross-sectional survey was conducted among the Libyan population between July 18 and August 23, 2020. The United Nations estimated that as of July 1, 2020, Libya had a population of 6,871,292 people.

### Sample Size and Sampling Technique

The sample size was determined using the single proportion formula, taking into account 53.8% of the population proportion value from the previous study conducted in China (13).

The sample size was calculated using the proportion formula of a cross-sectional study design (*n* = required sample size *n* = Z (α/2) 2 pq/d2), which was used for the calculations. Therefore, to calculate the required sample size, the size of the Libyan population (i.e., 6,871,292) was used, *p* = 0.54, q (1 - p) = 0.46, 95% CI, and a 1% sampling error was used. Here, *n* = required sample size, *n* = Z (α/2) 2 pp./d2, *p* = 0.54. This resulted in the required sample size of 9,530. Furthermore, as the study was based on an online survey, we may not have been able to estimate the response rate accurately, in order to add it to the sample size used in this study. The sampling technique involved a convenient sampling method. Respondents were free to answer the questions based on their participation desire. No type of reinforcement was used to encourage participants to answer questions, so as to avoid any potential bias.

### Study Participants and Data Collection Procedures

The survey was carried out using the online Google forms that was distributed among the Libyan population using emails, mobile messages, and social media. The survey included members of the Libyan population who had resided in Libya for at least 4 years and who did not leave Libya during the COVID-19 pandemic. To avoid any selection bias, fundamental questions, such as nationality, residency, and general questions about Libya were asked to ensure that the participants were from Libya. It was mandatory to complete all primary data variables required in the survey to avoid missing or incomplete data in the analysis. The online cross-sectional study was conducted in accordance with the Checklist for Reporting Results of Internet E-Surveys (CHERRIES) (37). The reporting of the study followed the statement outlined in *Strengthening the Reporting of Observational Studies in Epidemiology* (38).

### Instruments of the Study

The survey was conducted anonymously without identifiable data and it contained several sections as follows:

The first section included questions regarding basic demographic characteristics, such as gender, age, marital status, educational qualifications, occupation, stable or unstable income, a question about experiencing financial problems during the pandemic, and participants' current health status. A five-point Likert-type scale was used. Possible responses included “the best of health,” “good health,” “average health,” “I feel unwell,” and “I am very ill.”

The second section included questions related to the COVID-19 pandemic which covered the following: COVID-19 infection status, work status during the pandemic, presence of psychological, emotional, financial, or domestic violence issues during the lockdown, and lockdown compliance.

The third section included questions related to an assessment of the possibility of Internet overuse/misuse during the pandemic; the main reason for using the Internet; reasons for being unable to stop using the Internet despite its negative effects, such as neck and/or back pain after prolonged Internet use; Internet use causing sleep disturbances; the effect of the Internet on work responsibilities; negative feelings about using the Internet; whether or not the Internet was a form of escapism or if it interfered with social interactions; effects of the Internet on interactions with family or friends; and questions about reducing online Internet use.

The fourth section included questions related to the assessment of lifestyle and sleep changes during the pandemic lockdown. The questions covered the following: weight changes; attitudes about whether the pandemic affected sleep; sleep time before and after the pandemic; the amount of time needed to fall sleep before and after the pandemic; wake-up time before and during the pandemic; quality of sleep during the pandemic; presence of daytime sleepiness during the pandemic; and whether or not medication was taken for sleep.

The fifth section included the evaluation of insomnia, depression, and anxiety disorders during the pandemic. The Insomnia Severity Index (ISI) is a brief instrument that is used to assess the severity of both daytime and nocturnal episodes of insomnia. The instrument was validated by several previous studies in different languages, and it was deemed a valid and sensitive metric tool for sleep research to quantify the perceived severity of insomnia (39–42). The scores ranged from 0 to 28, where 0–7 indicated no clinically significant insomnia; 8–14, sub-threshold insomnia; 15–21, clinical insomnia (moderate severity); and 22–28, clinical insomnia (severe). The seven-item Generalized Anxiety Disorder scale (GAD-7) (43) was then used to detect anxiety symptoms, the total scores of which ranged from 0 to 21. The possible responses to each question included the following: “not at all” = 0, “several days” = 1, “more than half of the days” = 2, and “nearly every day” = 3. A cut-off score of ≥ 15 was proposed as indicative of anxiety symptoms (44, 45). The two-item Patient Health Questionnaire (PHQ-2) was used to measure depressive symptoms. Symptoms were evaluated by referring to the DSM-IV criteria for depression (46), which was validated by several studies (47–49). A total score ranged from 0 to 6. A cut-off score of ≥ 3 or more was deemed to indicate depressive symptoms (48, 50). Study tool can be found in English and Arabic language in Supplementary Files 1, 2.

### Data Quality Control

The English version of the questionnaires, which included the psychometric tools (i.e., PHQ-2, GAD-7, and ISI instruments) were translated into the Arabic language by two independent translators, and Arabic is the official language of the Libyan population. The questionnaires were then back-translated into English to determine the internal consistency of the English versions. Any discrepancy in the translations was resolved by a third person until a consensus was reached. Before the formal study was carried out, we conducted a pilot study involving 30 respondents to ensure a high level of internal consistency, as determined by a Cronbach's alpha value of above 0.7. The reported internal consistency of the PHQ-2 was determined by a Cronbach's alpha value of 0.828, while the GAD-7 had a Cronbach's alpha of 0.92. The ISI had a Cronbach's alpha value of 0.823. The first author trained data collectors over a 2-day period before the commencement of the study to ensure that they were aware of the study's process and to avoid any misunderstanding or errors in data collection. The authors then checked all of the data to ensure its quality by discarding any duplicate responses, thereby safeguarding the highest possible data quality for analysis.

### Statistical Analysis

Frequencies and percentages were used for the descriptive statistics of the variables. For continuous variables, the mean and standard deviation (SD) were used. A chi-square test was used to determine the association between the study variables and the PHQ-2, GAD7, and ISI grades. Binomial logistic regression was used to ascertain the effects of the study variables on the likelihood that participants will experience depressive (PHQ-2 ≥ 3) or non-depressive (PHQ-2 <3) symptoms, or the effects of the study variables on the likelihood that participants will experience anxiety symptoms (GAD-7 ≥ 15) or be less likely to experience anxiety symptoms (GAD-7 <15). In respect to the ISI score, binomial logistic regression was used to determine the effects of study characteristics on the probability that participants will be highly likely to suffer from clinical insomnia (ISI score 15–28) or less likely to suffer from clinical insomnia (ISI score <15). Nagelkerke *R*-Square was used to calculate the explained variation. Omnibus tests of the model coefficients were used to detect the overall statistical significance of the prediction models. A Wilcoxon signed-rank test was conducted to determine the effect of the COVID-19 pandemic on participants' changes in sleep time and wake-up time. The significance level was set at 0.05. Statistical analyses were performed using IBM SPSS Statistics version 25.0 for Windows.

### Ethical Considerations

The study was approved by the bioethics committee at the Biotechnology Research Centre in Libya. Participants provided their informed written consent before completing the anonymous survey, and no identifiable patient data were recorded.

## Results

A total of 10,296 responses were obtained from among the Libyan population, and data related to more than 20 cities. In this study, the majority of the respondents included 7,991 females (77.6%) and 2,305 males (22.4%). The mean (SD) age of the respondents was 28.9 (8.5). Most of the respondents were younger than 30 years old (6,657, 64.7%). Basic study characteristics, along with associated depressive and anxiety symptoms, are summarized in Table 1.

**Table 1:** Basic study characteristics and association with depressive and anxiety symptoms (*n* = 10,296).

**Characteristics**	**N (%)**	**Depressive symptoms**				**Anxiety symptoms**			
		**PHQ-2 ≥ 3** ***n* = 4,756**	**PHQ-2 <3** ***n* = 5,540**	**χ^**2**^**	***p*-value**	**GAD-7 ≥15** ***n* = 1,952**	**GAD-7 <15** ***n* = 8,344**	**χ^**2**^**	***p*-value**
**Gender**				0.71	0.4			2.27	0.132
- Male	2,305 (22.4)	1,047 (15)	1,258 (22.7)			412 (21.1)	1,893 (22.7%)		
- Female	7,991 (77.6)	3,709 (78)	4,282 (77.3)			1,540 (78.9)	6,451 (77.3%)		
**Age (years)**				277.6	<0.001^**^			146.22	<0.001^**^
- <30	6,657 (64.7)	3,478 (73.1)	3,179 (57.4)			1,492 (76.4)	5,165 (61.9%)		
- ≥ 30	3,639 (35.3)	1,278 (26.9)	2,361 (42.6)			460 (23.6)	3,179 (38.1%)		
**Marital status**				143.7	<0.001^**^			55.24	<0.001^**^
- Single (including widows and divorced)	6,594 (51)	3,337 (70.2)	3,257 (58.8)			1,392 (71.3)	5,202 (62.3%)		
- Married	3,702 (36)	1,419 (29.8)	2,283 (41.2)			560 (28.7)	3,142 (37.7%)		
**Highest education**				106.2	<0.001^**^			55.46	<0.001^**^
- Elementary	27 (0.3)	12 (0.3)	15 (0.3)			6 (0.3)	21 (0.3%)		
- Preparatory	261 (2.5)	137 (2.9)	124 (2.2)			64 (3.3)	197 (2.4%)		
- Secondary/diploma	2,570 (18)	1,296 (27.2)	1,274 (16)			556 (28.5)	2,014 (24.1%)		
- Bachelor/higher degree	6,393 (62.1)	2,975 (62.6)	3,418 (61.7)			1,205 (61.7)	5,188 (62.2%)		
- Post graduate (master, PhD)	857 (8.3)	279 (5.9)	578 (10.4)			104 (5.3)	753 (9%)		
- Other	188 (1.8)	57 (1.2)	131 (2.4)			17 (0.9)	171 (2%)		
**Occupation**				258.3	<0.001^**^			108.35	<0.001^**^
- Full-time employee	2,794 (27.1)	1,093 (16)	1,701 (30.7)			404 (20.7)	2,390 (28.6%)		
- Part-time employee	1,160 (11.3)	483 (10.2)	677 (12.2)			184 (9.4)	976 (11.7%)		
- Student	3,878 (37.7)	2,107 (44.3)	1,771 (32)			878 (45)	3,000 (36%)		
- Unemployed	2,118 (20.6)	996 (20.9)	1,122 (20.3)			454 (23.3)	1,664 (19.9%)		
- Retired	187 (1.8)	28 (0.6)	159 (2.9)			15 (0.8)	172 (2.1%)		
- Other	159 (1.5)	49 (22)	110 (23)			17 (0.9)	142 (1.7%)		
**Financial status**				111.3	<0.001^**^			66.88	<0.001^**^
- Stable income	4,012 (39)	1,593 (33.5)	2,419 (43.7)			602 (30.8)	3,410 (40.9%)		
- Non-stable income	6,284 (52)	3,163 (66.5)	3,121 (56.3)			1,350 (69.2)	4,934 (59.1%)		
**Financial issue during COVID-19 pandemic**				296.3	<0.001^**^			128.65	<0.001^**^
- Yes	5,145 (50)	2,812 (59.1)	2,333 (42.1)			1,201 (61.5)	3,944 (47.3%)		
- No	5,151 (50)	1,944 (40.9)	3,207 (57.9)			751 (38.5)	4400 (52.7%)		
**COVID-19 infection**				266.9	<0.001^**^			93.86	<0.001^**^
- Yes	1,525 (14.8)	998 (14)	527 (9.5)			426 (21.8)	1,099 (13.2%)		
- No	8,771 (85.2)	3,758 (79)	5,013 (90.5)			1,526 (78.2)	7,245 (86.8%)		
**Current health status**				538.7	<0.001^**^			370.54	<0.001^**^
- Best health	6,680 (64.9)	2,601 (54.7)	4,079 (73.6)			1,034 (53)	5,646 (67.7%)		
- Good health	1,943 (18.9)	1,001 (14)	942 (10)			375 (19.2)	1,568 (18.8%)		
- Average health	1,065 (10.3)	685 (14.4)	380 (6.9)			287 (14.7)	778 (9.3%)		
- I feels unwell	357 (3.5)	276 (5.8)	81 (1.5)			113 (5.8)	244 (2.9%)		
- I am very ill	251 (2.4)	193 (4.1)	58 (22)			143 (7.3)	108 (1.3%)		
**Considered suicide during COVID-19 lockdown**				751.9	<0.001^**^			596.98	<0.001^**^
- Yes	1,002 (9.7)	874 (18.4)	128 (2.3)			478 (24.5)	524 (6.3%)		
- No	9,294 (90.3)	3,882 (81.6)	5,412 (97.7)			1,474 (75.5)	7,820 (93.7%)		
**Abuse or domestic violence**				958.3	<0.001^**^			573.19	<0.001^**^
- Yes	3,579 (34.8)	2,399 (50.4)	1,180 (21.3)			1,132 (54)	2,447 (29.3%)		
- No	6,717 (65.2)	2,357 (49.6)	4,360 (78.7)			820 (42)	5,897 (70.7%)		
**Lockdown compliance**				130.7	<0.001^**^			64.21	<0.001^**^
- Fully compliant to instruction	2,551 (24.8)	965 (20.3)	1,586 (28.6)			443 (22.7)	2,108 (25.3%)		
- Most of the time	3,052 (29.6)	1,391 (29.2)	1,661 (30)			538 (27.6)	2,514 (30.1%)		
- Somewhat	2,197 (21.3)	1,141 (17)	1,056 (19.1)			418 (21.4)	1,779 (21.3%)		
- A little	1,475 (14.3)	703 (14.8)	772 (13.9)			266 (13.6)	1,209 (14.5%)		
- No personal isolation practice	1,021 (9.9)	556 (11.7)	465 (8.4)			287 (14.7)	734 (8.8%)		

*Significant at p < 0.05.

** *Significant at p < 0.001*.

The depressive symptoms, which were assessed using the PHQ-2 scale, had a mean (SD) score of 2.55 (1.9), and 4,756 respondents (46.2%) reached the cut-off score of ≥ 3 for depressive symptoms. In respect to anxiety, which was assessed using the GAD-7 score, the mean (SD) was 9.1 (5.9), and the following categories were identified: Minimal (0–4) 2,655 (25.8%); Mild (1, 2, 26–28) 3,088 (30%); Moderate (3–7) 2,601 (25.3%); and Severe (8–14) 1,952 (19%). For the ISI, the mean (SD) was 11.4 (6.1), and the following categories were identified: No clinical insomnia (0–7) 3,132 (30.4%); sub-threshold insomnia (1–7) 3,747 (36.4%); moderate severity clinical insomnia (8–14) 2,929 (28.4%); and severe clinical insomnia (15–21) 488 (4.7%).

A statistically significant association was found between depressive symptoms and the following variables: age range (under or over 30 years old), marital status, education level, occupational category, stable financial status, financial problems during the COVID-19 pandemic, COVID-19 infection status, current health status, suicide ideation during the pandemic, presence of abuse or domestic violence, and lockdown compliance (PHQ-2 ≥ 3, *p* < 0.05). However, no significant association was found between gender and depressive symptoms.

As shown in Table 1, a statistically significant association was found between anxiety symptoms and the following variables: age range (under or over 30 years old), marital status, education level, occupation, financial status and a stable income, financial difficulties, COVID-19 infection status, health status, suicide ideation, abuse or domestic violence, and lockdown compliance (GAD-7 ≥ 15, *p* < 0.05). Gender was the only variable that showed no statistically significant association with anxiety symptoms.

Binomial logistic regression was performed to ascertain the effects of the study variables on the likelihood that participants had depressive symptoms (PHQ-2 ≥ 3). The logistic regression model was statistically significant, $\chi_{19}^{2}$ = 2335.39, *p* < 0.001. The model explained 27.1% (Nagelkerke *R*^2^) of the variance in depressive symptoms and correctly classified 69.6% of cases. Sensitivity was 59.7%, specificity was 78.1%, the positive predictive value was 70.1%, and the negative predictive value was 69.3%. Of the study predictor variables, all of them were statistically significant except for gender and financial status (as shown in Table 2). Increasing age was associated with a reduction in the likelihood of exhibiting depressive symptoms, while married persons were 1.24 times more likely to exhibit depressive symptoms compare to single persons. A higher education level was associated with a higher likelihood of depressive symptoms, and bachelor degree holders were 2.17 times more likely to exhibit depressive symptoms, whilst post-graduate degree holders or students were 2.42 times more likely to exhibit depressive symptoms. The occupational category was associated with a lower likelihood of depressive symptoms, compared to fulltime employees. Retirement was associated with a reduction in the likelihood of depressive symptoms. However, having financial issues during the COVID-19 pandemic had 1.5 higher odds to exhibit depressive symptoms, being infected had 1.5 higher odds of depressive symptoms, suicidal ideation had 5.21 higher likelihood of depressive symptoms, and exposure to domestic violence or abuse had 2.66 higher likelihood to exhibit depressive symptoms. Those who reported feeling unwell or very ill were 3.13 and 4.7 times more likely to exhibit depressive symptoms, respectively. While respondents who abided by few isolation measures were 1.63 times more likely to exhibit depressive symptoms.

**Table 2:** Logistic regression: predicting the likelihood of depressive symptoms (PHQ-2 ≥ 3) based on study characteristics.

**Variable**	**B**	**SE**	**Wald**	***df***	***p-value***	**Odds ratio**	**95% CI for odds ratio**	
							**Lower**	**Upper**
**Age (years)**	0.025	0.004	41.42	1	<0.001^*^	0.97	0.96	0.98
**Gender**								
- Male						1		
- Female	0.036	0.059	0.378	1	0.53	1.04	0.92	1.16
**Marital status**								
- Single (including widows and divorced)					1			
- Married	0.215	0.06	12.959	1	<0.001^*^	1.24	1.103	1.394
**Highest education**								
- Elementary			35.34	5	<0.001^*^			
- Preparatory	0.66	0.501	1.73	1	0.187	1.94	0.73	5.17
- Secondary/diploma	0.96	0.236	16.75	1	<0.001^*^	2.63	1.65	4.17
- Bachelor/higher degree	0.77	0.195	15.73	1	<0.001^*^	2.17	1.48	3.18
- Post graduate (Master, PhD)	0.88	0.192	21.32	1	<0.001^*^	2.42	1.66	3.53
- Other	0.56	0.209	7.18	1	0.007	1.75	1.16	2.63
**Occupation**								
- Full-time employee			41.11	5	<0.001^*^			
- Unemployed	0.291	0.076	14.59	1	<0.001^*^	0.748	0.644	0.868
- Part-time employee	0.317	0.088	13.04	1	<0.001^*^	0.728	0.613	0.865
- Student	0.065	0.07	0.85	1	0.354	0.937	0.818	1.075
- Retired	1.178	0.258	20.87	1	<0.001^*^	0.308	0.186	0.51
- Other	0.577	0.196	8.66	1	0.003^*^	0.562	0.382	0.825
**Financial status**								
- Stable income						1		
- Non-stable income	0.034	0.057	0.359	1	0.549	1.035	0.926	1.156
Financial issue during COVID-19 pandemic	0.425	0.047	82.549	1	<0.001^*^	1.529	1.395	1.67
COVID-19 infection	0.43	0.67	41.546	1	<0.001^*^	1.538	1.349	1.75
**Current health status**								
- Best health			228.99	4	<0.001^*^			
- Good health	0.366	0.058	40.16	1	<0.001^*^	1.442	1.288	1.615
- Average health	0.713	0.076	87.62	1	<0.001^*^	2.041	1.757	2.369
- I feels unwell	1.143	0.144	63.15	1	<0.001^*^	3.135	2.365	4.156
- I am very ill	1.549	0.62	90.87	1	<0.001^*^	4.706	3.423	6.471
Considered suicide during COVID-19 lockdown	1.657	0.103	256.71	1	<0.001^*^	5.21	4.28	6.42
Abuse or domestic violence	0.98	0.049	401.07	1	<0.001^*^	2.66	2.42	2.93
**Lockdown compliance**								
- No personal isolation practices			51.36	4	<0.001^*^			
**-** A little	0.491	0.086	32.53	1	<0.001^*^	1.635	1.38	1.93
- Somewhat	0.352	0.076	21.25	1	<0.001^*^	1.422	1.23	1.65
- Most of the time	0.411	0.68	36.59	1	<0.001^*^	1.51	1.32	1.72
**-** Fully compliant to instruction	0.261	0.62	17.86	1	<0.001^*^	1.29	1.15	1.46

* Significant at p < 0.05. **Significant at p < 0.001.

A GAD-7 score of 15 or more was deemed indicative of anxiety symptoms. Binomial logistic regression was performed to determine the effects of the study variables on the likelihood that participants had anxiety symptoms (GAD-7 ≥ 15). The logistic regression model was statistically significant, $\chi_{19}^{2}$ = 1263.66, *p* < 0.001. The model explained 18.6% (Nagelkerke *R*^2^) of the variance with regard to anxiety symptoms and it correctly classified 82.3% of cases. Sensitivity was 19.6%, specificity was 96.9%, the positive predictive value was 60.03%, and the negative predictive value was 83.7%. Of the study characteristics, all of them were statistically significant except: gender, marital status, financial status, and lockdown compliance status (as shown in Table 3).

**Table 3:** Logistic regression predicting the likelihood of anxiety symptoms (GAD-7 ≥ 15) based on the study characteristics.

**Variable**	**B**	**SE**	**Wald**	***df***	***p*-value**	**Odds Ratio**	**95% CI for odds ratio**	
							**Lower**	**Upper**
**Age (years)**	0.024	0.005	23.018	1	<0.001^**^	0.97	0.96	0.98
**Gender**								
- Male						1		
- Female	0.068	0.073	0.873	1	0.35	1.07	0.92	1.23
**Marital status**								
- Single (including widows and divorced)						1		
- Married	0.061	0.076	0.658	1	0.42	1.06	0.92	1.23
**Highest education**								
- Elementary			22.31	5	<0.001^**^			
- Preparatory	1.195	0.592	4.07	1	0.04^*^	3.30	1.04	10.55
- Secondary/diploma	1.346	0.322	17.43	1	<0.001^**^	3.84	2.04	7.22
- Bachelor/higher degree	1.083	0.286	14.34	1	<0.001^**^	2.95	1.68	5.17
- Post graduate (Master, PhD)	1.123	0.283	15.74	1	<0.001^**^	3.07	1.76	5.35
- Other	0.868	0.304	8.16	1	0.004^*^	2.38	1.31	4.32
**Occupation**								
- Full-time employee			21.84	5	0.001^*^			
- Unemployed	0.338	0.094	12.87	1	<0.001^**^	0.71	0.59	0.85
- Part-time employee	0.369	0.11	11.34	1	0.001^*^	0.69	0.55	0.85
- Student	0.242	0.083	8.46	1	0.004^*^	0.78	0.66	0.92
- Retired	0.642	0.305	4.42	1	0.035^*^	0.53	0.29	0.95
- Other	0.611	0.276	4.9	1	0.027^*^	0.54	0.32	0.93
**Financial status**								
- Stable income					1			
- Non-stable income	0.049	0.069	0.5	1	0.479	0.95	0.83	1.09
Financial issue during COVID-19 pandemic	0.282	0.058	23.67	1	<0.001^**^	1.33	1.81	1.49
COVID-19 infection	0.207	0.073	8.017	1	0.005^*^	1.23	1.06	1.42
**Current health status**								
- Best health			186.626	4	<0.001^**^			
- Good health	0.14	0.072	3.79	1	0.051	1.15	0.99	1.33
- Average health	0.38	0.084	21.09	1	<0.001^**^	1.46	1.24	1.73
- I feels unwell	0.39	0.134	8.77	1	0.003^*^	1.48	1.14	1.93
- I am very ill	1.88	0.144	172.02	1	<0.001^**^	6.57	4.95	8.7
Considered suicide during COVID-19 lockdown	1.16	0.078	222.07	1	<0.001^**^	3.19	2.74	3.72
Abuse or domestic violence	0.93	0.058	256.31	1	<0.001^**^	2.52	2.56	2.83
**Lockdown compliance**								
- No personal isolation practices			23.02	4	<0.001^**^			
**-** A little	0.365	0.098	13.85	1	<0.001^**^	1.44	1.19	1.74
- Somewhat	0.034	0.094	0.13	1	0.72	0.96	0.8	1.16
- Most of the time	0.068	0.084	0.66	1	0.42	0.93	0.79	1.1
**-** Fully compliant to instruction	0.033	0.076	0.18	1	0.67	0.97	0.83	1.24

* *Significant at p <0.05*.

** *Significant at p <0.001*.

Increasing age was associated with a reduction in the likelihood of anxiety symptoms. Respondents with a higher educational level were more likely to exhibit anxiety symptoms. At the same time, for occupational status, there was a reduction in the likelihood of exhibiting anxiety symptoms according to each category of occupational type when compared to working as full-time employee during pandemic. Having financial issues during the COVID-19 pandemic had 1.33 times higher odds to exhibit anxiety symptoms, being infected with COVID-19 had 1.23 higher odds to exhibit anxiety symptoms, having suicidal ideation had 3.19 times higher likelihood of anxiety symptoms, and exposing to domestic violence or abuse during the pandemic had 2.52 higher likelihood to exhibit anxiety symptoms. Being unwell or very ill were both associated with an increase in anxiety symptoms by 1.48 and 6.57 times compared to being in best health status, respectively.

Additionally, a binomial logistic regression was performed to ascertain the effects of study variables on the likelihood that participants have a degree of anxiety symptoms as demonstrated by GAD-7 ≥ 10. The logistic regression model was statistically significant, $\chi_{19}^{2}$ = 2429.49, *p* < 0.001. The model explained 28.2% (Nagelkerke *R*^2^) of the variance with regard to anxiety symptoms and it correctly classified 71.5% of cases. Sensitivity was 59.6%, specificity was 80.9%, the positive predictive value was 71.2%, and the negative predictive value was 71.6%. Of the study characteristics, the following variables were statistically significant: age (OR 0.98, 95% CI 0.97–0.99, *p* < 0.001), being infected with COVID-19 (OR 1.55, 95% CI 1.36–1.77, *p* < 0.001), suicidal ideation (OR 6.12, 95% CI 4.97–7.53, *p* < 0.001), being exposed to the violence of abuse (OR 3.17, 95% CI 2.88–3.49, *p* < 0.001), and having financial issues during the pandemic (OR 1.8, 95% CI 1.64–1.97, *p* < 0.001).

A chi-square test was conducted between the study variables and the grades specified in the ISI, as illustrated in Table 4. All expected cell frequencies were greater than five. In addition, there was a statistically significant association between the four grades of the ISI and all of the study characteristics including gender, age range (under or over 30 years old), marital status, occupational status, financial status, financial status during the COVID-19 pandemic, COVID-19 infection status, health status, suicide ideation, abuse and/or domestic violence, and lockdown compliance. A strong association was found, as shown in Table 4.

**Table 4:** Study characteristics and the association with ISI grades (*n* = 10,296).

**Characteristics**	**N (%)**	**Insomnia severity index grades**					
		**No clinically significant insomnia (ISI score = 0–7)** ***n* = 3,132**	**Subthreshold insomnia (ISI score = 8–14)** ***n* = 3,747**	**Clinical insomnia (moderate severity) (ISI score = 15–21)** ***n* = 2,929**	**Clinical insomnia (severe) (ISI score = 22–28)** ***n* = 488**	**χ^**2**^**	***p*-value**
**Gender**						23.3	<0.001**
- Male	2,305 (22.4)	626 (13)	871 (23.2)	717 (24.5)	91 (18.6)		
- Female	7,991 (77.6)	2,506 (80)	2,876 (76.8)	2,212 (75.5)	397 (81.4)		
**Age (years)**						153.7	<0.001**
- <30	6,657 (64.7)	1,781 (56.9)	2,449 (65.4)	2,051 (70)	376 (77)		
- ≥ 30	3,639 (35.3)	1,351 (43.1)	1,298 (34.6)	878 (30)	112 (16)		
**Marital status**						78.2	<0.001**
- Single (including widows and divorced)	6,594 (51)	1,880 (55)	2,342 (62.5)	2,003 (68.4)	369 (75.6)		
- Married	3,702 (36)	1,252 (40)	1,405 (37.5)	926 (31.6)	119 (24.4)		
**Highest education**						72.7	<0.001**
- Elementary	27 (0.3)	9 (0.3)	9 (0.2)	9 (0.3)	0 (0)		
- Preparatory	261 (2.5)	75 (2.4)	121 (3.2)	54 (1.8)	11 (2.3)		
- Secondary/diploma	2,570 (18)	745 (23.8)	879 (23.5)	823 (28.1)	123 (25.2)		
- Bachelor/higher degree	6,393 (62.1)	1,918 (61.2)	2,421 (64.6)	1,740 (59.4)	314 (64.3)		
- Post graduate (Master, PhD)	857 (8.3)	303 (9.7)	272 (7.3)	247 (8.4)	35 (7.2)		
- Other	188 (1.8)	82 (2.6)	45 (1.2)	56 (1.9)	5 (22)		
**Occupation**						288.7	<0.001**
- Full-time employee	2,794 (27.1)	935 (29.9)	1,052 (28.1)	710 (24.2)	97 (19.9)		
- Part-time employee	1,160 (11.3)	365 (11.7)	421 (11.2)	329 (11.2)	45 (9.2)		
- Student	3,878 (37.7)	941 (30)	1,432 (38.2)	1,275 (43.5)	230 (47.1)		
- Unemployed	2,118 (20.6)	694 (22.2)	771 (20.6)	546 (18.6)	107 (21.9)		
- Retired	187 (1.8)	135 (4.3)	20 (0.5)	28 (22)	4 (0.8)		
- Other	159 (1.5)	62 (23)	51 (1.4)	41 (1.4)	5 (22)		
**Financial status**						111.1	<0.001**
- Stable income	4,012 (39)	1,410 (45)	1,482 (39.6)	987 (33.7)	133 (27.3)		
- Non-stable income	6,284 (52)	1,722 (56)	2,265 (60.4)	1,942 (66.3)	355 (72.7)		
**Financial issue during COVID-19 pandemic**						555.8	<0.001**
- Yes	5,145 (50)	1,084 (34.6)	1,892 (50.5)	1,826 (62.3)	343 (70.3)		
- No	5,151 (50)	2,048 (65.4)	1,855 (49.5)	1,103 (37.7)	145 (29.7)		
**COVID-19 infection**						273.4	<0.001**
- Yes	1,525 (14.8)	229 (7.3)	550 (14.7)	636 (21.7)	110 (22.5)		
- No	8,771 (85.2)	2,903 (92.7)	3,197 (85.3)	2,293 (78.3)	378 (77.5)		
**Current health status**						526.5	<0.001**
- Best health	6,680 (64.9)	2,417 (77.2)	2,377 (63.4)	1,613 (55.1)	273 (55.9)		
- Good health	1,943 (18.9)	454 (14.5)	795 (21.2)	612 (20.9)	82 (16.8)		
- Average health	1,065 (10.3)	129 (4.1)	395 (10.5)	451 (15.4)	90 (18.4)		
- I feels unwell	357 (3.5)	36 (1.1)	115 (3.1)	181 (6.2)	25 (5.1)		
- I am very ill	251 (2.4)	96 (3.1)	65 (1.7)	72 (2.5)	18 (3.7)		
**Considered suicide during COVID-19 lockdown**						657.6	<0.001**
- Yes	1,002 (9.7)	47 (1.5)	279 (7.4)	574 (19.6)	102 (20.9)		
- No	9,294 (90.3)	3,085 (98.5)	3,468 (92.6)	2,355 (80.4)	386 (79.1)		
**Abuse or domestic violence**						1054.5	<0.001**
- Yes	3,579 (34.8)	504 (16.1)	1,236 (33)	1,532 (52.3)	307 (62.9)		
- No	6,717 (65.2)	2,628 (83.9)	2,511 (57)	1,397 (47.7)	181 (37.1)		
**Lockdown compliance**						107.2	<0.001**
- Fully compliant to instruction	2,551 (24.8)	866 (27.7)	810 (21.6)	746 (25.5)	129 (26.4)		
- Most of the time	3,052 (29.6)	923 (29.5)	1,160 (31)	814 (27.8)	155 (31.8)		
- Somewhat	2,197 (21.3)	524 (16.7)	903 (24.1)	679 (23.2)	91 (18.6)		
- A little	1,475 (14.3)	480 (15.3)	541 (14.4)	407 (13.9)	47 (9.6)		
- No personal isolation practice	1,021 (9.9)	339 (10.8)	333 (8.9)	283 (9.7)	66 (13.5)		

Binomial logistic regression was performed to ascertain the effects of the basic study variables on the likelihood that the study's participants had clinical insomnia (ISI score 15–28) or no clinical insomnia (ISI score <15). We found that 3,417 (33.2%) participants had clinical insomnia vs. 6,879 (66.8%) participants who did not have clinical insomnia. The logistic regression model was statistically significant, $\chi_{19}^{2}$ = 1569.28, *p* < 0.001. The model explained 19.6% (Nagelkerke *R*^2^) of the variance in clinical insomnia and correctly classified 72.2% of cases. Sensitivity was 36.9%, specificity was 89.8%, the positive predictive value was 64.21%, and the negative predictive value was 74.1%. Of the study variables, all of them were statistically significant except age, education level, and occupation status (as shown in Table 5). Female had 1.28 times higher odds to exhibit insomnia compared to male, while married persons were 1.18 times higher to exhibit insomnia. Similar to previous outcomes, insomnia exhibit more for those with the following variables: 1.72 times higher among those with financial issues during the COVID-19 pandemic, 1.46 times higher with those infected with COVID-19, 2.76 times higher with those considered suicide or have suicidal ideation, and 2.42 times higher among those who exposed to abuse or domestic violence during the pandemic. Health status during the pandemic was associated with higher odds to exhibit insomnia with those with reported being ill or very ill had higher odds to exhibit insomnia.

**Table 5:** Logistic regression predicting the likelihood of insomnia severity (ISI score 15–28) based on the study characteristics.

**Variable**	**B**	**SE**	**Wald**	***df***	***p*-value**	**Odds ratio**	**95% CI for odds ratio**	
							**Lower**	**Upper**
**Age (years)**	0.007	0.004	3.16	1	0.07	0.99	0.98	1.001
**Gender**								
- Male						1		
- Female	0.247	0.06	17.09	1	<0.001^**^	1.28	1.13	1.44
**Marital status**								
- Single (including widows and divorced)						1		
- Married	0.169	0.062	7.44	1	0.006^*^	1.18	1.05	1.33
**Highest education**								
- Elementary			24.18	5	<0.001^**^			
- Preparatory	0.131	0.486	0.069	1	0.79	0.87	0.33	2.32
- Secondary/diploma	0.586	0.237	6.13	1	0.013	0.55	0.35	0.88
- Bachelor/higher degree	0.008	0.185	0.002	1	0.96	0.99	0.69	1.42
- Post graduate (Master, PhD)	0.053	0.181	0.085	1	0.77	1.05	0.74	1.5
- Other	0.277	0.198	1.96	1	0.16	1.32	0.89	1.94
**Occupation**								
- Full-time employee			13.894	5	0.016			
- Unemployed	0.096	0.079	1.46	1	0.225	0.91	0.77	1.06
- Part-time employee	0.059	0.09	0.423	1	0.515	0.94	0.79	1.13
- Student	0.134	0.07	3.48	1	0.06	1.14	0.99	1.32
- Retired	0.48	0.231	4.32	1	0.038	0.62	0.39	0.97
- Other	0.025	0.19	0.016	1	0.898	1.03	0.7	1.5
**Financial status**								
- Stable income						1		
- Non-stable income	0.17	0.058	8.91	1	0.003^*^	0.84	0.75	0.94
Financial issue during COVID-19 pandemic	0.54	0.048	127.48	1	<0.001^**^	1.72	1.56	1.88
COVID-19 infection	0.38	0.06	35.22	1	<0.001^**^	1.46	1.29	1.65
**Current health status**								
- Best health			117.46	4	<0.001^**^			
- Good health	0.24	0.059	16.77	1	<0.001^**^	1.27	1.14	1.43
- Average health	0.702	0.073	91.92	1	<0.001^**^	2.02	1.74	2.32
- I feels unwell	0.736	0.124	35.124	1	<0.001^**^	2.08	1.64	2.66
- I am very ill	0.308	0.148	4.33	1	0.038^*^	1.36	1.02	1.82
Considered suicide during COVID-19 lockdown	1.018	0.078	169.75	1	<0.001^**^	2.76	2.37	3.23
Abuse or domestic violence	0.88	0.048	331.23	1	<0.001^**^	2.42	2.19	2.65
**Lockdown compliance**								
- No personal isolation practices			23.94	4	<0.001^**^			
**-** A little	0.177	0.088	4.077	1	0.04^*^	0.84	0.71	0.99
- Somewhat	0.343	0.078	19.11	1	<0.001^**^	0.71	0.61	0.83
- Most of the time	0.209	0.069	9.24	1	0.002^*^	0.81	0.71	0.93
**-** Fully compliant to instruction	0.243	0.063	14.97	1	<0.001^**^	0.78	0.69	0.88

* *Significant at p <0.05*.

** *Significant at p <0.001*.

With regard to the assessment of Internet overuse or misuse during the COVID-19 pandemic, most of the participants (9,431; 91.6%) reported using the Internet for recreational purposes and/or for the purpose of contacting other people. However, 3,079 (29.9%) of the participants agreed that they could not stop using the Internet despite perceived negative effects. While 6,925 (67.3) participants believed that they used the Internet for longer than they had intended.

Among the participants, 5,368 (52.1%) reported neck or back pain due to prolonged Internet use. A total of 804 participants (7.8%) believed that Internet use may severely interfere with sleep, while 2,053 participants (19.9%) thought that Internet use interferes a lot with sleep. About half of the participants (4,553; 44.2%) reported tardiness in respect to work and other obligations that needed to be addressed due to excessive Internet use, while 1,608 participants (15.6%) reported that they felt angry about the amount of time that they spent on the Internet. More than half of the participants in the study (5,882; 57.1%) regarded the Internet as a form of escape that can be used to avoid situations and issues due to the COVID-19 pandemic, while 4,745 participants (46.1%) reported that their friends or family had complained to them about their excessive Internet use. Table 6 presents a summary of the assessment of participants' Internet use during the COVID-19 pandemic.

**Table 6:** Assessment of the possibility of internet overuse or misuse during the COVID-19 pandemic.

**Questions**	***n***	**%**
**1. What do you mostly use the internet for? (tick all that apply)**		
Work-related	8,752	85
Recreational and/or contacting other people	9,431	91.6
Educational	7,105	69
Other purposes	6,759	65.6
**2. I cannot stop using internet despite its negative effect on my life**		
Strongly agree	1,947	18.9
Agree	1,132	11
Neutral	3,306	32.1
Disagree	1,675	16.3
Strongly disagree	2,236	21.7
**3. Do you suffer from neck or back pain after prolonged internet use?**		
Yes	5,368	52.1
No	4,928	47.9
**4. Do you feel that you use the internet for more extended periods than you initially intend to?**		
Yes	6,925	67.3
No	3,371	32.7
**5. Does internet use interfere with your sleep?**		
Severely	804	7.8
A lot	2,053	19.9
Somewhat	3,655	35.5
Rarely	1,365	13.3
Not at all	2,419	23.5
**6. Have you been falling behind in your obligations, such as work or studies due to internet use?**		
Yes	4,553	44.2
No	5,743	55.8
**7. Do you feel angry with yourself over the time you spend online?**		
Most of the time	1,608	15.6
Sometimes	4,366	42.4
Rarely	1,623	15.8
Never	2,699	26.2
**8. Did you consider the internet as an escape from situations and problems due to the COVID-19 pandemic?**		
Yes	5,882	57.1
No	4,414	42.9
**9. Do you prefer internet use over social interaction?**		
Yes	4,746	46.1
No	5,550	53.9
**10. Have you had complained from family or friends regarding the amount of time you spent on the internet?**		
Yes	4,745	46.1
No	5,551	53.9
**11. Did you try to reduce the time that you spend on online activities?**		
Yes	4,847	47.1
No	5,449	52.9

In terms of changes in people's lifestyle and sleep patterns after the COVID-19 pandemic, 2,249 participants (21.8%) reported that they had gained weight during the pandemic. The participants in this study reported several factors that contributed to the quality of their sleep during the COVID-19 pandemic: Being unable to access medical care (6,972 participants; 67.7%) and having to put education plans on hold (6,564; 63.8%) were the major reasons reported. Among the participants, 7,327 (71.2%) reported daytime sleepiness during the pandemic, and 1,165 (11.3%) reported taking sleep medication to help them fall asleep. With regard to the overall quality of sleep during the pandemic, ~2,754 (26.7%) and 1,620 (15.7%) participants reported that the quality of the sleep was either worse or much worse, respectively. Table 7 shows the lifestyle and sleep changes reported by the participants in this study.

**Table 7:** Assessment of lifestyle and sleep changes after the COVID-19 pandemic.

**Questions**	***n***	**%**
**1. How has your body weight changed during the pandemic?**		
No change	3,522	34.2
Weight decrease	4,525	43.9
Weight increased	2,249	21.8
**2. Which effect of the pandemic do you think affected your sleep? (tick all that apply)**		
Being unable to go to work	4,585	44.5
Being forced to put your education on hold	6,564	63.8
Being unable to go to worship places	5,804	56.4
Being unable to visit friends and relatives or participate in social gatherings	4,670	45.4
Being unable to seek medical care	6,972	67.7
Other	5,607	54.5
**3. When do you usually go to bed (Before lockdown)**		
9 P.M. or before	868	8.4
10 P.M. to 1 A.M.	6,659	64.7
After 1 A.M.	2,769	26.9
**4. When do you usually wake up Before lockdown?**		
8 A.M. or before	3,336	32.4
8 A.M. to 12 P.M.	5,894	57.2
After 12 P.M.	1,066	10.4
**5. How long does it take for you to fall asleep before lockdown?**		
<15 min	3,234	31.4
15–30 min	5,733	55.7
30 min-1 h	1,329	12.9
**6. When do you usually go to bed during a lockdown?**		
9 P.M. or before	493	4.8
10 P.M. to 1 A.M.	3,335	32.4
After 1 A.M.	6,468	62.8
**7. When do you usually wake up during lockdown**		
8 A.M. or before	965	9.4
8 A.M. to 12 P.M.	5,290	51.4
After 12 P.M.	4,041	39.2
**8. How long does it take for you to fall asleep during COVID-19 lockdown?**		
<15 min	2,056	20
15–30 min	5,612	54.5
30 min-1 h	2,628	25.5
**9. How would you describe the overall quality of your sleep during the pandemic?**		
Much worse	1,620	15.7
Worse	2,754	26.7
No change	4,739	46
Better	895	8.7
Much better	288	2.8
**10. Did you start taking any medication to help you go to sleep after the pandemic?**		
Yes	1,165	11.3
No	9,131	88.7
**11. Did you suffer from daytime sleepiness during COVID-19 lockdown?**		
Yes	7,327	71.2
No	2,969	28.8

A Wilcoxon signed-rank test was conducted to determine the effect of COVID-19 on three main variables: time of going to bed before and during the COVID-19 pandemic, wake-up time before and during the COVID-19 pandemic, and the time needed to fall asleep before and during the COVID-19 pandemic. The difference rank scores were calculated. A total of 10,296 participants were recruited to take part in this study. There was a statistically significant median increase in the time at which participants went to bed before the pandemic compared to during pandemic, *z* = −51.85, *p* < 0.001. In addition, the pandemic was associated with a statistically significant median increase in participants' wake-up time before and during the pandemic, *z* = −56.16, *p* < 0.001. In respect to the amount of time needed to fall asleep, there was also a statistically significant median increase in the time it took to fall to asleep before the pandemic and during the pandemic, *z* = −38.52, *p* < 0.001.

## Discussion

Since the outbreak of the COVID-19 pandemic in December 2019, there have been extraordinary attempts by governments across the world to curtail its spread. These measures involved lockdowns, strong mitigation strategies, and economic shutdowns around the world. The exact effect of COVID-19 on mental well-being remains unclear and more studies are needed to assess this issue in more depth. Vulnerability in the area of mental well-being is generally perceived to be outweighed by a desire to avoid infection. The aim of our study was to establish the psychological status, sleep disturbances, and associated risk factors in a country that has been experiencing an ongoing civil war since 2011, in addition to the COVID-19 infectious disease pandemic, which peaked in July and August 2020.

This study offers insight into the Libyan population's current psychological status, sleep quality, Internet use, and the socioeconomic status during the COVID-19 pandemic. A rapidly disseminated survey allowed us to reach a large number of study participants, the number of which totaled 10,296 individuals in Libya. This study identified several factors that are associated with depressive and anxiety symptoms and insomnia. Approximately half of the population (46.2%) suffered from depressive symptoms, about one-fifth (19%) suffered from severe anxiety symptoms, and one-third (33.2%) had clinical insomnia as determined by the ISI scale. We also identified several factors that were associated with depression, anxiety symptoms, and insomnia. In addition, we found significant changes in sleep times and sleep patterns before and during the COVID-19 pandemic. We found a substantial increase in Internet use and misuse, as reported by the study's participants.

While 1,525 participants (14.8%) reported that they had developed COVID-19 symptoms, we found that 6,284 (61%) had a stable source of financial income, while 5,145 (50%) experienced financial problems during the COVID-19 pandemic. Moreover, we found that 1,002 participants (9.7%) contemplated suicide during the lockdown, and 3,579 (34.8%) reported abuse or domestic violence in their home during this period, which poses a significant concern. Logistic regression analysis revealed that a statistically significant association between depressive symptoms and age, marital status, education level, occupational category, financial problems during the COVID-19 pandemic, having a COVID-19 infection, current health status, suicide ideation, abuse or domestic violence, and lockdown compliance (*p* < 0.001). In respect to anxiety, the regression analysis showed a statistically significant association between anxiety symptoms and age, education level, occupational status, financial problems during the COVID-19 pandemic, having a COVID-19 infection, health status, suicide ideation, abuse and domestic violence, and lockdown compliance (*p* < 0.05). Therefore, these findings may also illustrate the possible detrimental psychological effects of the reported factors.

These results showed that many people are experiencing a higher than expected level of mental illness as well as socio-economic challenges, which further contributes to stress among these individuals. Suicide ideation, abuse and domestic violence having a COVID-19 infection, financial problems, and health status were associated with depressive and anxiety symptoms, as well as sleep disturbances. These findings highlight a concern about the mental health of the Libyan population.

Suicide has emerged as the COVID-19 pandemic spreading around the world, with recent reports of suicidal cases due to fear of contracting the virus or due to mental suffering that is not bearable (58). Previous suicidal cases in Pakistan and Bangladesh were reported in the literature (53, 59). Therefore, addressing the suicidal ideations and implementing effective mental health supporting strategies is crucial in order to avoid suicidal catastrophic consequences in Libya.

With regard to Internet use, approximately one-third of the participants were unable to stop using the Internet despite the negative effect that it had on their life. In comparison, 5,368 participants (52.1%) reported neck or back pain. Around half of the participants believed that the Internet interfered with their sleep, while approximately half of the participants (4,553; 44.2%) felt that they had fallen behind in their work or other obligations because of their Internet use. Approximately half of the participants (46.1%) preferred Internet use over social interactions, and they reported that they had received complaints from their family and friends. We also found a significant change in sleep time, wake-up time, and the time needed to fall asleep. Participants altered their sleep patterns and went to sleep at a later time during the pandemic than before the pandemic. Therefore, we expect that these findings can offer greater insight into the quality of sleep that people experienced during the crisis, which may have negative consequences in the long term.

The prevalence results of our study are higher than a previous study reported by Fu et al. (33) in Wuhan, China, where they found that 27.5% of participants had anxiety symptoms, 29.3% had depressive symptoms, and 30.0% had a sleep disorder. In addition, Ren et al. (60) found the prevalence of depressive at 18.8% and that of anxiety symptoms at 13.3% among 1,172 Chinese respondents (60). Another Chinese investigation reported that 8.0% have either moderate to severe anxiety using the GAD-7 scale and 7.4% have some degree of somatization (56). With the use of GAD-7 ≥ 5 to indicate anxiety symptoms and that of ISI ≥ 8 to indicate some degree of insomnia (61), found the prevalence of anxiety symptoms at 33.02% and that of insomnia at 24.66% from 125 Chinese cities' residents (62) found the prevalence of depressive symptoms at 47.6%, that of anxiety symptoms at 33.8%, and that of insomnia at 25.1% among the general population. Moreover, they found that of depressive symptoms at 57.6%, that of anxiety symptoms at 45.4%, and that of insomnia at 13% among healthcare workers. However, the Chinese studies mentioned above were mostly conducted in the early phase of the pandemic: between January and March, 2020. Therefore, the above findings showed their prevalence rates lower than the findings from our study, in which 7,641 (74.2%) have scored ≥ 5 on the GAD-7 scale and 7,164 (69.6%) scored ≥8 on the ISI scale.

In Italy, a cross-sectional study that was conducted in April 2020 involved 500 participants who reported a prevalence of 19.4% and 18.6% for mild and moderate-to-severe psychological distress (14). Another large-scale study involved 5,071 participants was carried out in Australia (16). The study found that 62% of participants reported depressive symptoms, 50% experienced anxiety symptoms, and 64% had elevated stress levels, the prevalence rates of which were higher than those in our study. However, they used the 21-item Depression Anxiety Stress Scales which is different from our tools. A systemic review of recent studies on COVID-19 included 28 articles and found anxiety and depression symptoms (16–28%). However, this systemic review included several letters to editors' articles, and it was not possible to conduct a formal systemic review or meta-analysis as intended.

The findings in relation to the psychological status of participants could be attributed to confusion resulting from the high risk of contagion associated with COVID-19 around the world. Other factors may include how people are being put into quarantine, which has affected their work, studies, and their normal life activities. The pandemic has also resulted in fewer socioeconomic and educational opportunities, which may have adversely affected people's attitudes and mental well-being because of their fears about the future. Another possible explanation is that the spreading of rumors and news about the disease, across the Internet, contributed to a deterioration in people's psychological health. The prolonged holidays, along with the socioeconomic stress that has arisen because of the COVID-19 pandemic, may have compromised people's ability to function, and this will increase anxiety, depression, and sleep disorders. Another major risk factor for the Libyan population is the civil war, which has negative consequences, such as the internal displacement of the population and a greater economic burden because many people have lost their homes or properties because of this civil war.

Equally responsive care on psychiatric issues, sleep disturbances, and socioeconomic support should be given to the Libyan people. However, psychiatric services in Libya have been severely affected by the civil war, and the main issue is the low number of psychiatric centers, which are limited to just a few centers in the main cities. A second issue involves the absence of a specialized training program for psychiatrists; there are no rehabilitation services for patients who suffer from any type of mental illness (54). According to Okasha et al. (63), there was a relatively low number of specialists in mental health in Libya, with 0.2 psychiatrists, 0.05 psychiatric nurses, and 1.5 social workers per 100,000 population. These gaps have led to a situation where there is extremely poor availability of human capital and mental health facilities.

Therefore, establishing comprehensive psychosocial and behavioral well-being resources, community treatment services, and trained psychiatric professionals can address the vital everyday requirements of psychologically disordered patients by offering useful advice about disease management to decrease the prevalence of psychological issues. Other countries provided psychological support hotlines to help the general population during this difficult time. At the same time, several psychological interventions were proposed for low- and middle-income countries (55). However, these interventions may pose several ongoing challenges, such as a scarcity of resources, access to mental health services, and diagnostic challenges during the COVID-19 pandemic. Healthcare providers may have to choose between diagnosing and commencing treatment of psychiatric patients, or offering supportive care during the COVID-19 pandemic (52), during which there is a shortage of psychopharmacological treatment. In addition, the provision of medication might pose a substantial challenge to such countries due to the cost of these drugs, which may also hinder the ability of patients to seek treatment (64). This presents yet another challenge for countries such as Libya, namely, the social stigma associated with psychiatric illness and discrimination against patients (65). Such patients may delay obtaining a diagnosis and seeking treatment for their psychiatric illness out of fear of being discriminated against in society and the workplace. Therefore, there is a need to implement social interventions and provide support for these patients by carrying out an educational campaign targeted toward the general population, so as to help increase detection rates and to highlight the potentially catastrophic effects of delaying treatment and diagnosis.

The value of sleep is psychologically important. In our study, we found that 33.2% of participants suffered from clinical insomnia, as determined by the ISI scale. In addition, the binary logistic regression model revealed a statistically significant association between clinical insomnia and gender, marital status, financial source, financial issues during the COVID-19 pandemic, COVID-19 infection status, health status, suicide ideation, abuse or domestic violence, and lockdown status (*p* < 0.001). This level of clinical insomnia can be explained in terms of neural sleep pathways that are strongly associated with other neural pathways that control mood and essential brain functions (51). Similar to a previous study, our study found that gender may be an influencing factor. The sleep quality of people and their mental health condition communicate with each other. Sleep problems contribute to poorer mental well-being, and the impact of sleep problems can have a significant impact on mood (66). We found that the time at which participants went to bed and woke up and the amount of time needed to fall asleep changed significantly when we compared pre- and post-pandemic sleep patterns. This finding is in consistent with the study carried out in Italy (29) which found that participants went to sleep at a later time and had a poorer quality of sleep during the pandemic. These sleep disturbances will be greater, especially among people who might suffer from anxiety and depression. Therefore, there is a need to identify coping mechanisms and increase the amount of exercise that people do during the lockdown, particularly as it was found that longer periods of physical exercise can induce the stimulation of the production of neurochemical mediators, such as endorphins, noradrenaline, serotonin, and dopamine which generate a feeling of calm and reduce sleep disturbances (57, 67, 68).

## Strengths and Limitations

This study carried out a survey of the psychological, behavioral, and sleep status of members of the Libyan population who were facing the COVID-19 pandemic. The study involved a relatively large sample size of 10,296 participants who were assessed using validated tools to detect insomnia as well as depressive and anxiety symptoms. The study identified several risk factors and carried out a reliable statistical analysis along with several questions using the proposed tool to provide an overview of Internet use and changes in sleep patterns. However, the study had several limitations that should be considered: First, no previous study was carried out the mental health of the Libyan population. Therefore, we cannot estimate the real effects of COVID-19, which may have resulted in a confounding bias with respect to the final results of the study, as Libya is experiencing an ongoing civil war. Second, the majority of the participants were women (77.6%), which might be because they were more motivated to participate in the study than males. Another limitation involved the use of a cross-sectional study design, which might make it difficult to draw conclusions. Thus, future cohort studies are needed. Another limitation of the study is that the majority of the participants were young people: 64.7% were under 30 years of age. Therefore, future studies should focus on older people in Libya. Finally, internet use and behavioral changes were explained descriptively without association with the three outcomes of interest (insomnia, anxiety, and depressive symptoms), which necessitate further validation and psychometric evaluation.

## Conclusion

COVID-19 had a major impact on psychological and sleep patterns among the general population. Confronted with the COVID-19 outbreak, the Libyan population showed high levels of psychological stress, such as depressive and anxiety symptoms. One-third of the Libyan population is suffering from clinical insomnia. Therefore, to address this crisis, policymakers need to devise effective measures to reduce mental health issues among people and improve their quality of life by providing socio-economic support and developing interventions aimed at reducing the mental health burden.

## Data Availability Statement

The raw data supporting the conclusions of this article will be made available by the authors, without undue reservation.

## Ethics Statement

The studies involving human participants were reviewed and approved by the bioethics committee at the Biotechnology Research Centre in Libya. The patients/participants provided their written informed consent to participate in this study.

## Author Contributions

ME conceptualized the study design, supervised the project, analyzed the data, and wrote the first draft of the manuscript. ME, AAlso, AM, and AZ contributed to the survey design and translation. All authors contributed to data collection, participated in revising the manuscript, all agreed to accept equal responsibility for the accuracy of this paper, and approved the final article.

## Conflict of Interest

The authors declare that the research was conducted in the absence of any commercial or financial relationships that could be construed as a potential conflict of interest.

## Supplementary Material

Supplementary File 1 English version of the questionnaire.

Supplementary File 2 Arabic version of the questionnaire.
