# Supplementary material for: Psychological Health, Sleep Quality, Behavior, and Internet Use Among People During the COVID-19 Pandemic: A Cross-Sectional Study
Source: Front Psychiatry. 2021 Mar 31;12:632496. doi: 10.3389/fpsyt.2021.632496 (PMC8044819; doi:10.3389/fpsyt.2021.632496)
Supplement: Supplementary File 1 — English version of the questionnaire. [file Table_1.DOCX]

***Sleep Quality, Psychological Health, and Internet Use During COVID-19***

*As a result of the recent spread of Corona virus and the exceptional situation we are living in, we are trying to understand how people are adapting to the restrictions imposed upon our daily life, This survey shall be part of a statistical study aimed at evaluating the main changes that occurred in your daily life during the Pandemic, especially mental health, internet use & sleep disturbance .*

*We are a group of doctors and volunteer students seeking to mitigate the negative effects on our society by understanding the effects of this recent pandemic on our community.*

*We ask that you fill out this data, which may take 10 minutes of your time, this questionnaire is anonymous and no personal information is required, the data collected will be used purely for statistical purposes and will remain confidential and anonymous, to be used for statistical purposes only, we ask that you give your answers with complete honesty to show how affected you are by the Corona pandemic.*

***Completion of this questionnaire is deemed as an approval consent by your esteemed person to participate in the research. The results of the research may be the subject of publications.***

- **Gender**
- Male
- Female
- Age in Years: - ___________________________
  **Marital status:**
- Single
- Married
- **Nationality**
- Libyan Citizen
- Non-Libyan
- **Highest Educational Qualification**
- Elementary Certificate
- Preparatory Stage Completion Certificate
- Secondary Stage Completion Certificate
- Bachelor Degree / Higher Education
- Post Graduate (Master, PhD)
- None of the above
- **Occupation**
- Unemployed
- Full-Time Employee
- Freelance
- Student
- Retired
- Other
- Are you a healthcare worker or medical student?
- Healthcare worker
- Medical Student
- No
- Financial status
- Stable income
- Non-stable income
- Did you suffer Financial problems during the pandemic?
- Yes
- No

***Part 1 : COVID-19 Pandemic***

- How would you describe your level of compliance with the conditions of isolation and the safe distance imposed by the Local authorities to face the Corona pandemic ?
- No, I do not practice any kind of personal isolation
- A little
- Sometimes
- Most of the time
- Fully compliant to lockdown instructions
- During the Pandemic did you develop symptoms consistent with COVID-19 infection (fever, runny nose, cough, headache, sore throat, breathing difficulty)
- Yes
- No
- Current Health status :
- The best of health
- Good Health
- Average Health
- I feel unwell
- I am very ill
- Work status after the Corona pandemic
- The work situation has not changed
- The work load has increased
- The modus operandi changed (remote work, for example)
- I am not currently working
- How often do you find yourself feeling bored & having nothing to do during lockdown ?
- Never
- Rarely
- Sometimes
- Most of the time
- Almost always
- How has your body weight changed during the pandemic ?
- It increased
- No change
- It decreased.
- Have you had any of the following problems during the pandemic period? (More than one answer can be selected)
- Depression
- Abuse or domestic violence
- Emotional problems
- Financial problems
- periods of anxiety and stress
- Seriously considered suicide

***Part 2: Internet Use***

- Average no. of hours you use the Internet every day ______________________
- What do you mostly use internet for? (tick all that apply)
- Work related
- Recreational
- Contacting other people
- Educational
- Other purposes
- Do you suffer from Neck or back pain after prolonged internet use?
- Yes
- No
- Do you feel that you use the internet for longer periods than you originally intend to?
- Yes
- No
- Have you been falling behind in your obligations such as work or studies due to internet use?
- Yes
- No
- Do you spend more time online than you spend with your family?
- Yes
- No
- Do you prefer internet use over social interaction with friends or family?
- Yes
- No
- Have you had complained from family or friends regarding the amount of time you spend online?
- Yes
- No
- Do you consider the internet an escape from daily situations& personal problems?
- Yes
- No
- Do you feel excited about going online or checking your messages?
- Yes
- No
- Does internet use interfere with your sleep?
- Not at all
- Rarely
- Somewhat
- A lot
- Severely
- Have you failed before in reducing your online activity time?
- Yes
- No
- Do you feel angry with yourself over the time you spend online?
- Never
- Rarely
- Sometimes
- Most of the time
- Always
- I Cannot stop using internet despite its negative effect on my life
- Does not apply
- Agree
- Strongly Agree
- Disagree
- Strongly Disagree
- Do you think your life can be better if you reduce your internet use?
- Yes
- No

***Part 3 : Changes in Sleep pattern***

| When do you usually go to bed ? | | | | | | |
| --- | --- | --- | --- | --- | --- | --- |
| Before Lock Down | 9 pm or before | 10 pm | 11pm | 12pm | 1 am | after 1 am |
| Partial Lock Down (12-18h) | 9 pm or before | 10 pm | 11pm | 12pm | 1 am | after 1 am |
| Full Lockdown (24h) | 9 pm or before | 10 pm | 11pm | 12pm | 1 am | after 1 am |
| When do you usually wake up ? | | | | | | |
| Before Lock Down | 5 am or before | 6 am | 7 am | 8 am | 9 am | after 9 am |
| Partial Lock Down (12-18h) | 5 am or before | 6 am | 7 am | 8 am | 9 am | after 9 am |
| Full Lockdown (24h) | 5 am or before | 6 am | 7 am | 8 am | 9 am | after 9 am |

| How long does it take for you to fall asleep ? | | | | |
| --- | --- | --- | --- | --- |
| Before Lock Down | <15 min | 15-30 min | 30 min-1h | more than 1 hour |
| During Lockdown | <15 min | 15-30 min | 30 min-1h | more than 1 hour |

- Which effect of the pandemic do you think affected your sleep ? (tick all that apply)
- Being unable to go to work
- Being forced to put your education on hold
- Being unable to go to the mosque
- Being unable to visit friends and relatives or participate in social gatherings
- Being unable to seek medical care
- Being forced to stay indoors
- Other
- Did you suffer from daytime sleepiness during lockdown ?
- Yes
- No
- How would you describe the overall quality of your sleep during the pandemic ?
- Much worse
- Worse
- No change
- Better
- Much better
- Did you start taking any medication to help you go to sleep after the pandemic ?
- Yes
- No

***Part 4 : Mental Health***

| How many of you have experienced the following problems during the past month (tick the most appropriate answer) | | | | |
| --- | --- | --- | --- | --- |
| Feeling nervous, anxious or on edge? | | | | |
| - Not at all | - Several Days | - More than Half the days | - Nearly every day | |
| Not being able to stop or control worrying? | | | | |
| - Not at all | - Several Days | - More than Half the days | | - Nearly every day |
| Worrying too much about different things? | | | | |
| - Not at all | - Several Days | - More than Half the days | | - Nearly every day |
| Trouble relaxing? | | | | |
| - Not at all | - Several Days | - More than Half the days | | - Nearly every day |
| Being so restless that it is hard to sit still? | | | | |
| - Not at all | - Several Days | - More than Half the days | | - Nearly every day |
| Becoming easily annoyed or irritable? | | | | |
| - Not at all | - Several Days | - More than Half the days | | - Nearly every day |
| Feeling afraid as if something awful might happen? | | | | |
| - Not at all | - Several Days | - More than Half the days | | - Nearly every day |

*Over the last 2 weeks, how often have you been bothered by any of the following problems?*

| Little interest or pleasure in doing things | | | | |
| --- | --- | --- | --- | --- |
| - Not at all | - Several Days | - More than Half the days | - Nearly every day | |
| Feeling down, depressed, or hopeless | | | | |
| - Not at all | - Several Days | - More than Half the days | | - Nearly every day |

For each question, please choose the answer you deem most appropriate.

*Please rate the CURRENT (i.e. LAST 2 WEEKS) SEVERITY of your insomnia problem(s) (if any).*

| **Insomnia Problem** | **None** | **Mild** | **Moderate** | **Severe** | **Very Severe** |
| --- | --- | --- | --- | --- | --- |
| 1. Difficulty falling asleep |  |  |  |  |  |
| 1. Difficulty staying asleep |  |  |  |  |  |
| 1. Problems waking up too early |  |  |  |  |  |

4. How SATISFIED/DISSATISFIED are you with your CURRENT sleep pattern?

- Very Satisfied
- Satisfied
- Moderately Satisfied
- Dissatisfied
- Very Dissatisfied

5. How NOTICEABLE to others do you think your sleep problem is in terms of impairing the quality of your life?

- Not at all Noticeable
- A Little
- Somewhat
- Much
- Very Much Noticeable

6. How WORRIED/DISTRESSED are you about your current sleep problem?

- Not at all
- Worried A Little
- Somewhat
- Much
- Very Much Worried

7. To what extent do you consider your sleep problem to INTERFERE with your daily functioning (e.g. daytime fatigue, mood, ability to function at work/daily chores, concentration, memory, etc.) CURRENTLY?

- Not at all Interfering
- A Little
- Somewhat
- Much
- Very Much Interfering
