# Supplementary material for: Psychological Health, Sleep Quality, Behavior, and Internet Use Among People During the COVID-19 Pandemic: A Cross-Sectional Study
Source: Front Psychiatry. 2021 Mar 31;12:632496. doi: 10.3389/fpsyt.2021.632496 (PMC8044819; doi:10.3389/fpsyt.2021.632496)
Supplement: Supplementary File 2 — Arabic version of the questionnaire. [file Table_2.DOCX]

**مشاكل النوم والإكتئاب والقلق واستعمال الإنترنت في ظل جائحة كوفيد 19**

في أثناء الجائحة الحالية لفيروس كورونا والوضع الاستثنائي الذي نعيش فيه ، نحاول أن نفهم كيف يتكيف المجتمع مع القيود المفروضة على حياتنا اليومية ، سيكون هذا الاستطلاع جزءًا من دراسة إحصائية تهدف إلى تقييم التغييرات الرئيسية التي حدثت في الحياة اليومية أثناء الوباء ، وخاصة الصحة العقلية ، واستخدام الإنترنت واضطرابات النوم وعلاقتها بالجائحة.

نحن مجموعة من الأطباء والطلاب المتطوعين الذين يسعون لتخفيف الآثار السلبية على مجتمعنا من خلال فهم آثار هذا الوباء على مجتمعنا.

نطلب منك تعبئة هذه البيانات ، والتي قد تستغرق 10 دقائق من وقتك ، هذا الاستبيان لا يحمل أي معلومات شخصية ، وسيتم استخدام البيانات التي يتم جمعها فقط لأغراض إحصائية وستبقى سرية ومجهولة المصدر ، ليتم استخدامها لأغراض إحصائية فقط ، نطلب منك أن تقدم إجاباتك بأمانة تامة لإظهار مدى تأثرك أثناء جائحة كورونا.

يعتبر إكمال هذا الاستبيان بمثابة موافقة من قبلكم للمشاركة في البحث. قد تكون نتائج البحث موضوع نشر في المستقبل.

- **الجنس**
- ذكر
- أنثى
- **العمر بالسنوات**______________________
- الحالة الاجتماعية :
- أعزب
- متزوج
- **الجنسية :**
- ليبي
- غير ليبي
- **أعلى مؤهل تعليمي :**
- ابتدائي
- إعدادي
- ثانوي
- شهادة جامعية / معهد عالي
- دراسات عليا
- لا شيء مما سبق
- **الوظيفة الحالية :**
- عاطل عن العمل
- موظف بدوام كامل
- عمل حر
- طالب
- متقاعد
- أخرى
- **هل أنت تعمل في قطاع الصحة أو أنك طالب في كلية الطب ؟**
- أعمل في قطاع الصحة
- طالب في كلية الطب
- لا شيء مما سبق
- **الوضع المادي :**
- دخل ثابت
- دخل غير ثابت
- هل عانيت من مشاكل مادية خلال الجائحة ؟
- نعم
- لا

الجزء الأول : جائحة فيروس كورونا 2019

كيف تصف مستوي تقيدك بشروط العزل وتطبيق مبدأ المسافة الآمنة الذي تفرضه السلطات المحلية لمكافحة جائحة كورونا ؟

لا, لا أمارس أي نوع من العزل الشخصي

قليلا

بعض الأحيان

أغلب الوقت

ملتزم تماما بشروط العزل

خلال الجائحة, هل ظهرت عليك أعراض تتفق مع عدوي فيروس كورونا 2019 (ارتفاع درجة الحرارة, سيلان الأنف, سعال, صداع, التهاب حلق, صعوبة في التنفس)

نعم

لا

الوضع الصحي الحالي :

بأفضل صحة

صحة جيدة

صحة عادية

أشعر بتوعك بسيط

أشعر باعياء شديد

وضع العمل بعد جائحة كورونا

لم يتغير وضع العمل

ازداد ضغط العمل

طريقة العمل تغيرت (عمل عن بعد علي سبيل المثال)

لا أعمل حاليا

هل تشعر بالملل خلال فترة الحظر وأنك لا تجد ما تفعل؟

لا, أبدا

نادرا

أحيانا

أغلب الوقت

دائما

كيف تغير وزنك خلال الجائحة ؟

ازدادوزني

لم يتغير وزني

نقص وزني

هل عانيت من أي من هذه المشاكل خلال فترة الجائحة ؟ (يمكن اختيار أكثر من إجابة)

اكتئاب

مشاكل عائلية وتعدي بالضرب او الشتم

مشاكل عاطفية

مشاكل مادية

فترات من القلق و الضغط النفسي

فكرت جديا بالانتحار

الجزء الثاني استخدام الانترنت

متوسط عدد الساعات التي تستخدم فيها الانترنت يوميا ______________________

ما هي استخداماتك للانترنت؟ (اختر كل ما ينطبق)

العمل وما يتعلق به

استخدام ترفيهي

الاتصال والتواصل

التعليم

استخدامات أخري

هل تعاني من ألام في الرقبة أو ألام في الظهر بعد الاستخدام المستمر للانترنت ؟

- نعم
- لا

هل تشعر أنك تستخدم الانترنت لفترات أطول مما تريد ؟

- نعم
- لا

هل تتأخر في تنفيذ واجباتك مثل العمل والدراسة بسبب استخدام الانترنت ؟

- نعم
- لا

هل تقضي فترات أطول علي الانترنت مما تقضي مع عائلتك ؟

- نعم
- لا

هل تفضل استخدام الانترنت علي التفاعل الاجتماعي مع أصدقائك أو عائلتك ؟

- نعم
- لا

هل تلقيت انتقادات من عائلتك أو أصدقائك تتعلق بكمية الوقت الذي تقضيه علي الانترنت ؟

- نعم
- لا

هل تعتبر استخدام الانترنت بمثابة ملاذ للهروب من المواقف اليومية والمشاكل الشخصية ؟

- نعم
- لا

هل تتحمس لفكرة دخولك علي الانترنت أو تفقد بريد رسائلك ؟

- نعم
- لا

هل يؤثر استخدامك للانترنت سلبا علي انتظامك في النوم ؟

- لا أبدا
- نادرا
- أحيانا
- كثيرا
- دائما

هل فشلت من قبل في تقليل استخدامك للانترنت؟

- نعم
- لا

هل تشعر بالاستياء من نفسك بخصوص الوقت الذي تقضيه في استخدام الانترنت ؟

- لا أبدا
- نادرا
- أحيانا
- كثيرا
- دائما

لا أستطيع التوقف عن استخدام الانترنت رغم التأثير السلبي الموجود علي حياتي

أتفق بشدة

أتفق

لا ينطبق علي وضعي الحالي

لا أتفق

لا أتفق بشدة

هل تعتقد أن حياتك ستتغير للأفضل إذا قللت من استخدامك للانترنت ؟

- نعم
- لا

الجزء الثالث : التغير في نمط النوم

| **متي تخلد للنوم عادة ؟** | | | | | | |
| --- | --- | --- | --- | --- | --- | --- |
| **قبل الحظر** | 9 صباحا أو قبل ذلك | 10 صباحا | 11 صباحا | 12 صباحا | 1 ظهرا | بعد الواحدة ظهرا |
| **خلال الحظر الجزئي (12-18 ساعة)** | 9 صباحا أو قبل ذلك | 10 صباحا | 11 صباحا | 12 صباحا | 1 ظهرا | بعد الواحدة ظهرا |
| **خلال الحظر الكلي (24 ساعة)** | 9 صباحا أو قبل ذلك | 10 صباحا | 11 صباحا | 12 صباحا | 1 ظهرا | بعد الواحدة ظهرا |
| **متي تستيقظ عادة ؟** | | | | | | |
| **قبل الحظر** | 5 صباحا أو قبل ذلك | 6 صباحا | 7 صباحا | 8 صباحا | 9 صباحا | بعد 9 صباحا |
| **خلال الحظر الجزئي (12-18 ساعة)** | 5 صباحا أو قبل ذلك | 6 صباحا | 7 صباحا | 8 صباحا | 9 صباحا | بعد 9 صباحا |
| **خلال الحظر الكلي (24 ساعة)** | 9 صباحا أو قبل ذلك | 10 صباحا | 11 صباحا | 12 صباحا | 1 ظهرا | بعد الواحدة ظهرا |

| كم من الوقت تستغرق عادة لتخلد للنوم ؟ | | | | |
| --- | --- | --- | --- | --- |
| قبل الجائحة | أقل من 15 دقيقة | 15- 30 دقيقة | 30 دقيقة إلي ساعة | أكثر من ساعة |
| خلال الجائحة | أقل من 15 دقيقة | 15- 30 دقيقة | 30 دقيقة إلي ساعة | أكثر من ساعة |

أي من الأثار السلبية للجائحة تعتقد أنه أثر في قدرتك علي النوم ؟

عدم القدرة علي الذهاب للعمل

توقف الدراسة والبرنامج التعليمي

اقفال المساجد

عدم القدرة علي زيارة الأصدقاء والأقارب أو المشاركة في المناسبات الاجتماعية

عدم القدرة علي الاستفادة من الخدمات الصحية

اجباري علي البقاء داخل البيت

أسباب أخري

هل تشعر بالنعاس والرغبة في النوم أثناء ساعات النهار؟

- نعم
- لا

كيف تقيم طبيعة النوم خلال الجائحة ؟

أسوأ بكثير

أسوأ

لا تغيير

أفضل

أفضل بكثير

هل بدأت بتعاطي دواء معين يساعد علي النوم بعد الجائحة ؟

- نعم
- لا

الجزء الرابع : الصحة النفسية

| **كم عانيت من المشاكل التالية خلال الشهر الماضي (ضع علامة √ أمام الإجابة الأنسب)** | **أبدا** | **بعض الأيام** | **أغلب الأيام** | **كل الأيام تقريبا** |
| --- | --- | --- | --- | --- |
| الشعور بالغضب أو القلق أو الانفعال الشديد |  |  |  |  |
| عدم القدرة على إنهاء القلق أو التحكّم فيه |  |  |  |  |
| القلق المفرط على أشياء مختلفة |  |  |  |  |
| الصعوبة في الاسترخاء |  |  |  |  |
| شدة الاضطراب لدرجة صعوبة البقاء ساكنا |  |  |  |  |
| السرعة في الانزعاج أو الانفعال |  |  |  |  |
| الشّعور بالخوف كما لو أن شيئا سيئا قد يحدث |  |  |  |  |

| خلال الأسبوعين الماضيين, كم عانيت مما يلي | **أبدا** | **بعض الأيام** | **أغلب الأيام** | **كل الأيام تقريبا** |
| --- | --- | --- | --- | --- |
| قلة الاهتمام أو عدم إيجاد سعادة عند انجاز المهام |  |  |  |  |
| الإحساس بالاكتئاب أو اليأس أو الإحباط |  |  |  |  |

لكل سؤال , اختر الإجابة الأنسب.

كيف تقيم مشاكل الأرق وصعوبة النوم خلال الأسبوعين الماضيين

| الأرق ومشاكل النوم | **أبدا** | **قليلا** | **بشكل معقول** | **كثيرا** | **كثيرا جدا** |
| --- | --- | --- | --- | --- | --- |
| صعوبة في الاستسلام للنوم |  |  |  |  |  |
| صعوبة في البقاء نائما |  |  |  |  |  |
| الاستيقاظ باكرا جدا |  |  |  |  |  |

إلي أي حد أنت راض عن نمط النوم الحالي لديك ؟

غير راضي أبدا

غير راضي

راضي قليلا

راضي

راض تماما

كم تعتقد أن الأشخاص حولك لاحظوا معاناتك من مشاكل النوم وتأثيرها علي حياتك؟

غير ملحوظ

قليلا

أحيانا

كثيرا

ملحوظ بشكل كبير

ما مستوي القلق لديك بخصوص مشاكل النوم التي تعاني منها ؟

لست قلقا

قلق قليلا

أقلق أحيانا

أقلق كثيرا

أقلق بشكل كبير

إلي أي حد تؤثر مشاكل النوم حاليا في مهامك اليومية (علي سبيل المثال الاعياء أثناء النهار, المزاج, القدرة علي انجازعملك , التركيز, الذاكرة) ؟

لا تؤثر

قليلا

بشكل ملحوظ

بشكل كبير

تأثير كبير جدا
